# Tuberculous Peritonitis

**Published:** 1897-12-11

**Authors:** E. Percy Paton

**Affiliations:** Surgical Registrar to Westminster Hospital, and Surgeon to the Mildmay Hospital


### Medical Progress and Hospital Clinics.

[*The Editor will be glad to receive offers of co-operation and contributions from members of the profession. All letters should be addressed to* The Editor, at the Office, 28 & 29, Southampton Street, Strand, London, W.C.]

### TUBERCULOUS PERITONITIS.

By E. Percy Paton, M.D., M.S.Lond., F.R.C.S., Surgical Registrar to Westminster Hospital, and Surgeon to the Mildmay Hospital.

(*Continued from page* 152.)

The complications of tuberculous peritonitis, apart from the tuberculous process becoming generalised or attacking other organs (especially the lungs and pleuræ), to which allusion has already been made, are especially due either to the matting by adhesions or to the breaking down and suppuration of the tuberculous deposit. The former not very infrequently gives rise to acute intestinal obstruction, due most commonly to the general adhesions preventing the movement of the intestines one upon another, so that, when one part becomes full, traction causes narrowing of another portion; in which case no cause for complete obstruction can be found at the autopsy. This cause may also give rise to attacks of incomplete obstruction, and in these cases even complete obstruction may be relieved without operative interference. Obstruction may also be caused by the formation of bands of adhesion producing internal strangulation. When suppuration occurs, it may be general throughout the whole peritoneal cavity or limited by adhesions. In the former case, if left alone, it ultimately most usually stretches the umbilicus and then discharges itself here. In the latter, as the pus increases, it tends to involve the skin which overlies it, and may ultimately discharge itself in this way, or by a similar process it may open into the intestine, or it may open in both directions at once, by which means a fæcal fistula is established. In any case of suppuration the tendency is distinctly in a downward direction, and if a sinus forms, as in tuberculous sinuses elsewhere, it is exceedingly difficult to get it to close, more especially as in most cases it is impossible to treat it satisfactorily.

The treatment of tuberculous peritonitis may be either medical or surgical. The medical means consist of treatment directed to the improvement of the general health by careful feeding and hygiene measures combined with rest. The patient should be confined to bed, but not necessarily kept absolutely in the house, provided the bed (or its equivalent) can be moved into the open air. Of course, the patient should only be taken out of doors when the weather is suitable, and he must be carefully protected from any danger from chills. The advantages, without the trouble, of moving out of doors can be obtained if the bed can be moved up to a sunny window, which can be opened when the weather is suitable. Fresh air and sunlight are often forgotten in the treatment of tubercle. The drugs usually given are cod-liver oil and iron in various forms, such as phosphate, iodide, &c. The oil, however, not infrequently cannot be continued, owing to the diarrhœa which it sometimes sets up in these cases. Locally, some recommend covering the abdomen with lint, on which is spread mercury ointment, or the use of a mercuric liniment, care being taken not to produce salivation. These medical means may be tried for a time; but should no improvement be speedily evident, operative measures should be undertaken. The simplest procedure is that of removal of the fluid by puncture; but it is not to be recommended, as, should there be adhesions, the main focus of fluid may be missed; or, worse still, the intestine may be punctured, owing to its adhesion to the anterior abdominal wall. It is better, therefore, to make an incision into the peritoneum, through which the fluid can be let out, and any adhesions which may prevent its escape can, if they are not too extensive, be broken down. The incision should practically always be made through the linea alba below the umbilicus. On exposing the peritoneum this may be found to be very thickened from tuberculous deposit, and not infrequently the omentum, and sometimes the gut, may be adherent to it, so that great care must be exercised in opening the cavity. The fluid should then be let out, and any which remains removed by sponges on holders passed into the flanks and pelvis. By some it is recommended to wash out the cavity with sterile water or boracic, but it is probably better not to do this. A little iodoform may be dusted in if desired, and the wound then closed without drainage. In many cases this simple procedure—the risk of which, if properly carried out, is very slight—has a most beneficial effect; the patient, whose condition has previously been practically stationary or slowly going down hill, may at once begin to improve in every respect. The above procedure is indicated in the ascitic cases and in fibrous cases in which the adhesions are not so extensive as absolutely to preclude any chance of opening any cavity. The proportion of cures in these cases is given by Treves as between 35 and 40 per cent. In the ulcerative cases operative interference, except for the purpose of draining collections of pus, is not to be recommended. If a fæcal fistula occurs, but little can usually be done except by scraping, and a plastic operation to close the skin-opening, which is not usually successful. Any more extensive interference is rendered hopeless owing to the way in which the coils of intestines are matted to one another. This same matting also renders very unsatisfactory any operative interference should obstruction occur, except when this is due to a simple band of adhesion.

